# Combination of traditional Chinese medicine and standard biomedical treatment for rosacea: a systematic review and a meta-analysis

**DOI:** 10.3389/fphar.2024.1397141

**Published:** 2024-08-08

**Authors:** Ruoxi Yu, Shuyan Li, Yuting Yang, Chunguang Xie, Qiu Chen, Ya Liu, Sihan Peng

**Affiliations:** ^1^ School of Clinical Medicine, Chengdu University of Traditional Chinese Medicine, Chengdu, China; ^2^ TCM Regulating Metabolic Diseases Key Laboratory of Sichuan Province, Hospital of Chengdu University of Traditional Chinese Medicine, Chengdu, Sichuan, China; ^3^ Hospital of Chengdu University of Traditional Chinese Medicine, Chengdu, Sichuan, China

**Keywords:** rosacea, acne rosacea, Chinese herbal medicine (CHM), combination of traditional Chinese medicine and standard biomedical Treatment, systematic review, meta-analysis

## Abstract

**Background:**

A combination of standard biomedical treatment and traditional Chinese medicine (TCM) has been suggested as a therapeutic approach for rosacea that may significantly lower the recurrence rate and clinical symptom scores. We conducted a systematic review and meta-analysis of randomized controlled trials (RCTs) to evaluate the impact of this combination treatment on clinical symptom and TCM syndrome scores, as well as on the scores of the Dermatology Life Quality Index (DLQI), erythema index (EI), and interleukin 37 (IL-37) levels in patients with rosacea.

**Methods:**

The PROSPERO registration number for the study is CRD42023472737. We systematically searched the PubMed, Embase, Web of Science, China National Knowledge Infrastructure Wanfang Database, China Biomedical Medicine database (CBM), and the VIP information resource integration service platform (cqvip) databases for RCTs (published from the beginning to September 2023, regardless of the language used) that compared the traditional Chinese medicine and standard biomedical treatment combination treatment to conventional anti-rosacea treatments. Our primary outcomes comprised the clinical symptom and TCM syndrome scores, and the scores of Dermatology Life Quality Index, erythema index, and IL-37 levels. We used a random-effects model to evaluate the pooled data.

**Results:**

We identified 260 studies. Of these, 13 eligible studies were employed for analysis (*N* = 1,348 participants). Compared with other anti-rosacea treatments, the TCM and standard biomedical treatment combination treatment yielded an improved mean reduction in the clinical symptom score −2.24% [95% CI (–3.02 to −1.46), *p* < 0.00001], TCM syndrome score −4.42 [95% CI (–5.33 to −3.50), *p* < 0.00001], and the score of DLQI of −2.55 [95% CI (–3.73 to −1.36), *p* < 0.00001], EI of −151.97 [95% CI (–276.59 to −27.36), *p* < 0.00001], and IL-37 level −4.23 [95% CI (–4.95 to −3.51), *p* = 0.854], as well as in the overall effective rate risk ratio (RR) = 1.25 [95%CI (1.18, 1.32), *p* = 0.994] and the recurrence rate = 0.27 [95%CI (0.15, 0.46), *p* = 0.297].

**Conclusion:**

The TCM and standard biomedical treatment combination treatment can provide a better outcome, including a reduction in the TCM syndrome and clinical symptom scores, and in the scores of DLQI, EI, and IL-37. Hence, this combination is a viable and more effective therapeutic approach for rosacea. However, these results should be considered cautiously because of uncertain evidence and the low quality of the study reports considered in this meta-analysis.

**Systematic Review Registration:**

website, identifier CRD42023472737.

## 1 Introduction

Rosacea is a long-term, inflammatory skin condition that often affected the central area of the face. It affects the facial blood vessels and sebaceous glands, causing vascular congestion, edema, dryness, papules, telangiectasia, and temporary flushing, apart from making the skin sensitive. (Anzengruber et al., 2017). Some patients may experience ocular symptoms following the onset of the disease, including a degree of excrescence. These uncomfortable symptoms could make the affected patient physically uncomfortable (owing to symptoms such as burning and stinging) and psychologically distressed. (Wollina, 2019). Many patients begin to exhibit psychological disorders such as depression and irritability owing to persistence of the disease, its recurrence, its unfavorable effect on facial appearance, and the resulting social isolation. However, there is still not much known about the factors responsible for rosacea. Vascular hyper-reactivity, immune system malfunction, and UV radiation exposure have been suggested as some of the possible factors that cause rosacea. (Zhang et al., 2021a). The pathophysiology and associated mechanisms are still under investigation, making it difficult to fully treat rosacea. Numerous psychiatric conditions, including sadness and anxiety, are also linked to rosacea. (Incel Uysal et al., 2019). Hence, it is essential to consider the mental health and overall quality of life of the patients while treating rosacea.

At present, the following four therapeutic approaches are used for the treatment of rosacea: medication, surgery, physical therapy, and general treatment. Metronidazole and clindamycin gels are typically used as local medications, while macrolides (such as isotretinoin soft capsules) and antibiotics (such as doxycycline and minocycline) are generally employed as systemic medications. The physical therapy mainly comprises red and blue light irradiation, laser therapy, and other techniques. However, the use of standard biomedical treatment causes several side effects, such as long-term recurrence rates and adverse clinical effects. (Steinhoff et al., 2016; Zip, 2017). In addition, standard biomedical treatment cannot adequately relieve the psychological feelings of patients. Therefore, it becomes essential to develop some alternative treatments, preferably those with greater efficacy and fewer adverse effects. The combination therapy comprising traditional Chinese medicine (TCM) and standard biomedical treatment is attracting increasing interest for the treatment of rosacea. Numerous clinical trials have demonstrated the efficacy of standard biomedical treatment and TCM combination treatments in lowering clinical symptom and TCM syndrome scores, as well as the scores of Dermatology Life Quality Index (DLQI), erythema index (EI), and interleukin 37 levels (IL-37). By using evidence-based techniques, we herein seek to systematically evaluate the safety and effectiveness of TCM and standard biomedical treatment combination therapy in the treatment of rosacea and to produce data supporting its clinical application.

## 2 Methods

### 2.1 Search strategy and selection criteria

The Preferred Reporting Items for Systematic Reviews and Meta-Analyses (PRISMA) Statement (Moher et al., 2009) is followed for the reporting of this systematic review and meta-analysis, which was filed at the International Prospective Register of Systematic Reviews (number CRD42023472737).

We thoroughly searched the PubMed, Web of Science, Embase, China National Knowledge Infrastructure (CNKI), China Biomedical Medicine database (CBM), Wanfang Database, and the VIP information resource integration service platform (cqvip) databases to identify related studies published between the start of the project and September 2023. We did not set up any linguistic limitations. The following combined text and MeSH words were employed: “Rosacea” [MeSH Terms] OR (“Rosacea” [MeSH Terms] OR “Rosacea” [All Fields] OR (“acne” [All Fields] AND “Rosacea” [All Fields]) OR “acne rosacea” [All Fields] OR (“Rosacea” [MeSH Terms] OR “Rosacea” [All Fields] OR (“phymatous” [All Fields] AND “Rosacea” [All Fields]) OR “phymatous rosacea” [All Fields]) OR (“Rosacea” [MeSH Terms] OR “Rosacea” [All Fields] OR (“Rosacea” [All Fields] AND “phymatous” [All Fields]) OR “rosacea phymatous” [All Fields]) OR (“Rosacea” [MeSH Terms] OR “Rosacea” [All Fields] OR (“ocular” [All Fields] AND “Rosacea” [All Fields]) OR “ocular rosacea” [All Fields]) OR (“Rosacea” [MeSH Terms] OR “Rosacea” [All Fields] OR (“Rosacea” [All Fields] AND “ocular” [All Fields]) OR “rosacea ocular” [All Fields]) OR (“Rosacea” [MeSH Terms] OR “Rosacea” [All Fields] OR (“papulopustular” [All Fields] AND “Rosacea” [All Fields]) OR “papulopustular rosacea” [All Fields]) OR (“Rosacea” [MeSH Terms] OR “Rosacea” [All Fields] OR (“Rosacea” [All Fields] AND “papulopustular” [All Fields]) OR “rosacea papulopustular” [All Fields]) OR (“Rosacea” [MeSH Terms] OR “Rosacea” [All Fields] OR (“erythematotelangiectatic” [All Fields] AND “Rosacea” [All Fields]) OR “erythematotelangiectatic rosacea” [All Fields]) OR (“Rosacea” [MeSH Terms] OR “Rosacea” [All Fields] OR (“Rosacea” [All Fields] AND “erythematotelangiectatic” [All Fields]) OR “rosacea erythematotelangiectatic” [All Fields]) OR (“Rosacea” [MeSH Terms] OR “Rosacea” [All Fields] OR (“granulomatous” [All Fields] AND “Rosacea” [All Fields]) OR “granulomatous rosacea” [All Fields]) OR (“Rosacea” [MeSH Terms] OR “Rosacea” [All Fields] OR (“Rosacea” [All Fields] AND “granulomatous” [All Fields]) OR “rosacea granulomatous” [All Fields]). We considered all potentially eligible studies for review, irrespective of the primary outcome or language.

### 2.2 Inclusion and exclusion criteria

The meta-analysis included all RCTs evaluating the effects of Chinese herbal medicine (CHM) on rosacea. The inclusion criteria were as follows: 1) The study should be an RCT. 2) Patients should have a confirmed diagnosis of rosacea without any restriction in terms of gender, nationality, ethnicity, or educational background. 3) Patients in the intervention group should have received CHM (including decoctions, pills, and granules, regardless of duration) along with standard biomedical treatment, and the control group should be treated with standard biomedical treatment in a similar manner as the intervention group. 4) The clinical symptom, TCM syndrome, and scores of DLQI scores should be the main results. The EI, IL-37 level, overall effective rate, side effects, and recurrence rate should be the secondary results. The exclusion criteria were as follows: 1) Non-RCT studies, including case reports, reviews, retrospective studies, animal experiments, and conference abstracts. 2) The use of additional TCM treatments, including massage, moxibustion, or acupuncture, on patients. 3) Research with insufficient information about the results.

### 2.3 Selection of studies and extraction of data

Two researchers (RY and SL) used the abovementioned inclusion and exclusion criteria to search and screen for relevant papers. Any disagreements were resolved through consensus. The EndNote V. X9 software was utilized to organize the literature. Using standardized extraction forms, two reviewers (RY and SL) independently extracted the data related to the first author, year of publication, country, sample size, gender, average age, duration of disease, interventions, adverse events, details about CHM (prescription name and composition), and outcomes from the eligible studies. YY verified the retrieved data. A third reviewer (SP) was on hand to settle any disagreements.

### 2.4 Bias risk assessment

By using the Risk of Bias tool of Cochrane Collaboration (Higgins et al., 2019), which includes blinding, selective reporting, random sequence generation, allocation concealment, inadequate data, and other biases, two reviewers (RY and YY) independently evaluated the risk of bias. Any differences arising between the two reviewers were arbitrated by another investigator (SP), who rated the results as “low,” “high,” or “unclear.”

### 2.5 Statistical analysis

The following five outcomes were used to evaluate the impact of TCM and standard biomedical treatment combination therapy: clinical symptom score; TCM syndrome score; and the scores of DLQI, EI, and IL-37 levels. We examined the scores of DLQI, EI, IL-37 levels, TCM syndrome scores, and clinical symptom scores as continuous variables and presented the absolute differences between arithmetic means before and following therapies.

We computed the overall relative risk across studies regarding the percentage of the overall efficacy rate, adverse effects, and recurrence rate. The overall effective rate was evaluated by considering the TCM syndrome score and/or the clinical symptom score. The efficacy index was rated as cured, marked response, response, or no response, and was determined using the following equation:

$$\text{Efficacy index}=\left(\text{total score before treatment }-\text{ total score after treatment}\right)/\text{total score before treatment}\times 100\%.$$

The total effective rate was calculated as follows:

Total effective rate=cured+marked response+response

The recurrence rate was calculated based on the follow-up results of the patients after the completion of the treatment course. Three (Mao and Wei, 2020; Xie, 2020; Li, 2021) of the 13 trials followed up with the patients after treatment. Two (Mao and Wei, 2020; Li, 2021) of these studies followed up the patients for 3 months after the completion of the treatment and the remaining study (Xie, 2020) followed up the patients for 6 weeks after the completion of the treatment course. The *X* ^2^ and *I* ^2^ tests were used to examine the heterogeneity of the data. If the data were homogeneous (*p* > 0.05, *I* ^2^ < 50%), a fixed effects model was utilized; otherwise, a random-effects model was employed (Higgins et al., 2003). Values with *p* < 0.05 were considered statistically significant. A subgroup analysis was conducted to examine the elements causing heterogeneity. We conducted pre-planned sensitivity analyses by focusing on studies that compared TCM and standard biomedical treatment combination treatments with conventional anti-rosacea therapies. The comparison of the relative roles of TCM and standard biomedical treatment helped reduce heterogeneity in treatment-induced changes in the outcomes of the overall analysis. We constructed a funnel plot of the effect size of each trial against the standard error to evaluate the potential for publication bias. The asymmetry of the funnel plot was determined using Egger tests. *p*-values of <0.1 indicated considerable publication bias. Each statistical analysis was conducted using Stata (version 18.0).

## 3 Outcomes

### 3.1 Choice of articles

**FIGURE 1:**
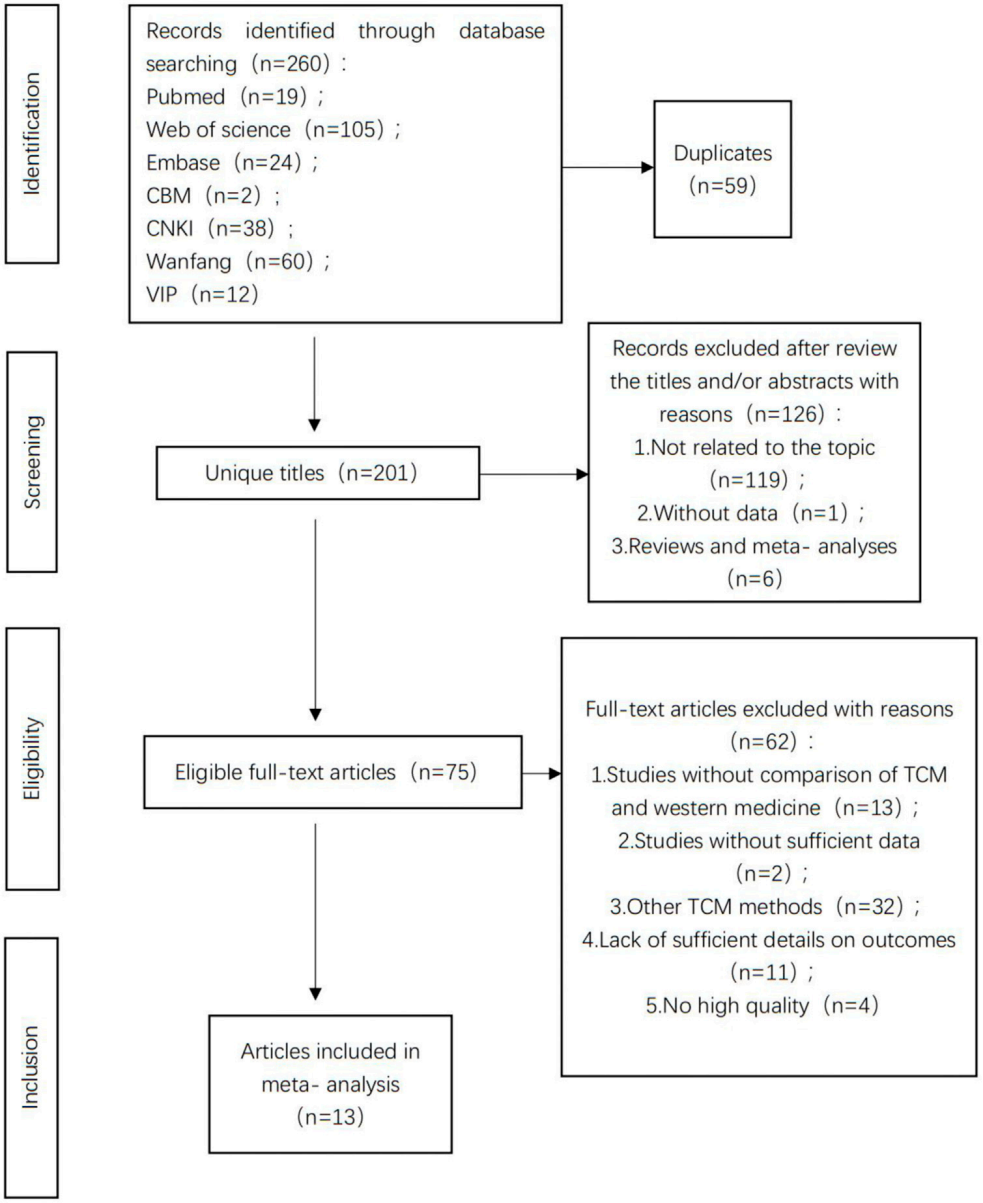
Study selection process.

Our search led to 260 studies. Of these, we used 13 studies (containing data on 1,348 participants) (Figure 1). We first assessed 260 pertinent articles identified using our search strategy. After eliminating all duplicate studies, we screened the remaining 201 articles based on their titles and abstracts. Next, we removed papers that did not fit our inclusion criteria (such as reviews and animal experiments), followed by an additional screening of the 75 articles. Among these 75 articles, we eliminated the following after conducting a thorough full-text review: 1) Lack of a comparison between TCM and standard biomedical treatment (*n* = 13); 2) insufficient data (n = 2); 3) use of other TCM techniques (*n* = 32); 4) insufficient details about the results (n = 11); 5) poor data quality (*n* = 4). We finally performed this meta-analysis using 13 publications (Wan et al., 2017; Xu et al., 2019; Mao and Wei, 2020; Wu, 2020; Xie, 2020; Li, 2021; Zhang et al., 2021b; Li et al., 2021; Yin, 2021; Xu and Chen, 2022; Yang, 2022; Fan et al., 2023; Liu et al., 2023).

### 3.2 Study characteristics

All 13 trials (Table 1) were published between 2017 and 2023. Each trial lasted an average of 6.62 weeks, with a range of 4–16 weeks. Nine of these 13 trials explicitly reported erythematotelangiectatic rosacea or papulopustular rosacea among the enrolled patients. From the diagnostic perspective of TCM, most of the studies tended to focus on the wind-heat of the lung meridian, damp heat of the spleen and stomach, stagnation of heat in the liver channel, and fire-type rosacea. In four trials, CHM was compared to a placebo as well as against a baseline treatment therapy comprising basic medication and antibiotics (Wan et al., 2017; Xu et al., 2019; Xie, 2020; Yin, 2021). Three studies compared CHM with a placebo and a laser treatment (Li, 2021; Fan et al., 2023; Liu et al., 2023). The remaining four trials compared the efficacy of CHM against a background of antibiotics, along with metronidazole gel and placebo medications (Zhang et al., 2021b; Li et al., 2021; Xu and Chen, 2022; Yang, 2022). In one study, topical treatment was used as a backdrop before comparing the CHM with a placebo (Li, 2021). By referring to the “type A extract” of the ConPhyMP consensus statement (Heinrich et al., 2022), we compiled a summary table (Supplementary Table S1) describing the botanical drug components and how they were reported in the original research.

**TABLE 1:** Characteristics of the included studies.

Study	Year	Sample size t/c	Age		Number of women		Duration of rosacea		Types of rosacea	Duration of interventions	Intervention (The composition of TCM is in Latin)		Outcomes
			T	C	T	C	T	C			T	C	
Mao and Wei (2020)	2020	55/54	38.19 ± 1.12	38.15 ± 1.09	43	40	5.28 ± 1.02	5.12 ± 1.08	NR	8w	hydroxychloroquine sulfate tablets + Rose Yurong decoction	hydroxychloroquine sulfate tablets	---
Xu et al. (2019)	2019	60/60			44	20			erythematotelangiectatic rosacea or papulopustular rosacea	8w	minocycline + Liangxue Xiaofeng San	minocycline	①
Wan et al. (2017)	2017	48/44	34.2 ± 2.6	34.8 ± 2.9	27	48	2.11 ± 0.42	2.14 ± 0.45	NR	8w	minocycline + liangxue qingfei power	minocycline	①
Yang (2022)	2022	30/30	35.56 ± 4.25	36.42 ± 4.29	21	27	2.61 ± 0.24	2.63 ± 0.55	NR		oral hydroxychloroquine and topical metronidazole liniment + Liangxuefei Decoction	oral hydroxychloroquine and topical metronidazole liniment	①④⑤
Xie (2020)	2020	32/32	37.59 ± 10.86	36.59 ± 10.04	28	20			erythematotelangiectatic rosacea or papulopustular rosacea	4w	Doxycycline + Jiawei-baihu decotion	doxycycline	①
Li (2021)	2021	32/31	35.81 ± 8.73	35.83 ± 8.59	23	27	16.78 ± 10.48	16.93 ± 9.70	NR	8w	red and blue light + Modified Feng Sui pellets	metronidazole + red and blue light	①⑤
Wu (2020)	2020	30/30	38.57 ± 10.68	39.67 ± 8.79	22	24	0.97 ± 0.55	0.86 ± 0.52	erythematotelangiectatic rosacea or papulopustular rosacea	4w	Compound Cortex Phellodendri liquid and Lvliu cream + Fuhe Decoction	Compound Cortex Phellodendri liquid and Lvliu cream + Tanshinone capsule	②③
Liu et al. (2023)	2023	60/60	26.35 ± 2.49	26.74 ± 2.53	32	27	1.56 ± 0.42	1.62 ± 0.45	NR	4w	+modified Chaishao Longmu Decoction	red and blue light	③
Fan et al. (2023)	2023	50/50	27.65 ± 5.43	30.11 ± 5.68	22	26	8.65 ± 1.43	8.48 ± 1.36	erythematotelangiectatic rosacea or papulopustular rosacea	4 m	intense pulsed light (IPL) +modified Qingre Chushi Decoction	intense pulsed light (IPL)	②③
Li et al. (2021)	2021	55/55	36.82 ± 2.09	36.24 ± 2.11	34	27	5.2 1 ± 1.03	5.12 ± 1.06	erythematotelangiectatic rosacea or papulopustular rosacea	6w	metronidazole gel and doxycycline hydro- chloride + Yiqi Yangyin Sanxie Decoction	metronidazole gel and doxycycline hydro- chloride	③
Zhang et al. (2021a)	2021	156/156	42.2 ± 7.3	42.1 ± 7.7	85	88	12.2 ± 3.2	12.1 ± 3.1	erythematotelangiectatic rosacea or papulopustular rosacea	4w	Doxycyclane Hyclate Tablets combined with Metronidazole Gel + *Liangxue-Qingfei* Decoction	Doxycyclane Hyclate Tablets combined with Metronidazole Gel	②④
Xu and Chen (2022)	2022	40/40	38.85 ± 5.20	38.96 ± 5.39	27	29	3.48 ± 0.59	3.59 ± 0.63	erythematotelangiectatic rosacea or papulopustular rosacea	4w	doxycycline hyclate combined with metronidazole + Liangxue Qingfei Decoction	doxycycline hyclate combined with metronidazole	②④
Yin (2021)	2021	29/29	40.38 ± 4.77	39.48 ± 4.55	16	15	2 2.7 1 ± 7.9 0	2 2.1 6 ± 8.6 6	erythematotelangiectatic rosacea or papulopustular rosacea	4w	Doxycycline hydrochloride enteric capsule + Huangqin Qingfei decoction	Doxycycline hydrochloride enteric capsule	③⑤

NR, not reported. ①the clinical symptom score; ②the scores of DLQI; ③TCM, syndrome scores; ④EI; ⑤IL-37.

TCM, traditional Chinese medicine; DLQI, dermatology life quality index; EI, erythema index; IL-37, interleukin 37 level.

CHM, chinese herbal medicine.

### 3.3 Risk of bias

**FIGURE 2:**
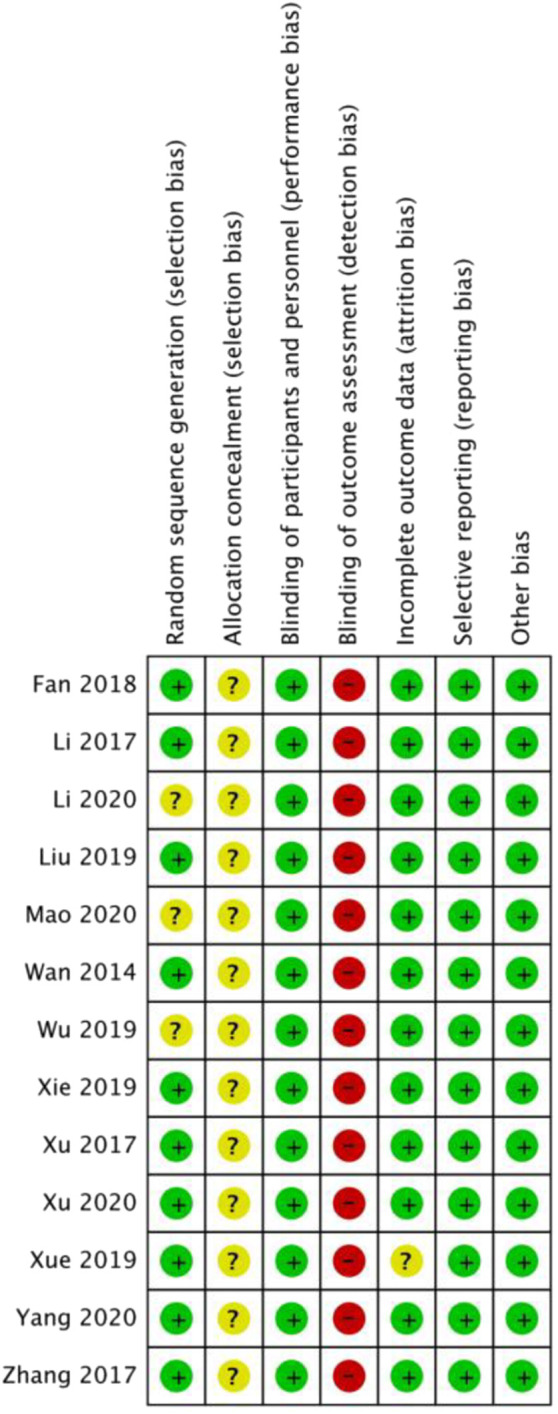
Risk of bias summary.

**FIGURE 3:**
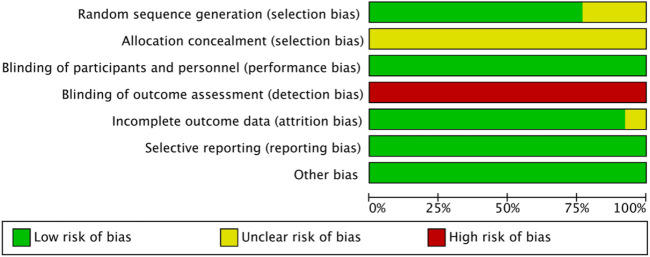
Risk of bias graph.

The outcomes of the risk of bias evaluation are displayed in Figure 2 and Figure 3. Because the included studies used a random number table for randomization, 10 of the 13 studies (Wan et al., 2017; Xu et al., 2019; Xie, 2020; Zhang et al., 2021b; Li et al., 2021; Yin, 2021; Xu and Chen, 2022; Yang, 2022; Fan et al., 2023; Liu et al., 2023) were categorized as having minimal risk of bias. The remaining three studies (Mao and Wei, 2020; Wu, 2020; Li, 2021) were labeled as “unclear risk” because they claimed to utilize randomization but did not disclose the specifics of the technique used for randomization. The allocation concealment was not evident in every study. None of the investigations reported the blinding of the participant or the researcher. Hence, we categorized every study as having an ambiguous risk of bias. Since none of the studies addressed the blinding of outcome assessment, this factor was deemed highly risky across all investigations. Each study that we included was rated as “low risk” in terms of additional biases.

### 3.4 Impact of CHM on the clinical symptom score

**FIGURE 4:**
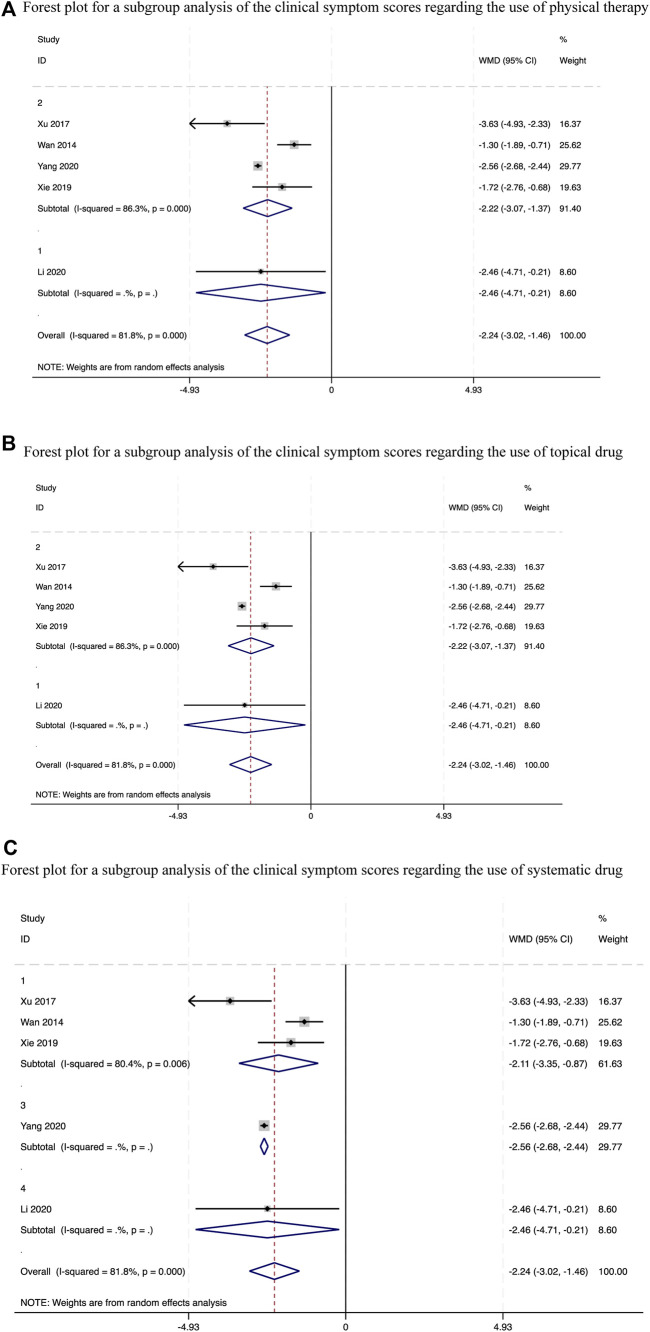
Forest plots for a subgroup analysis of the clinical symptom scores.

**FIGURE 5:**
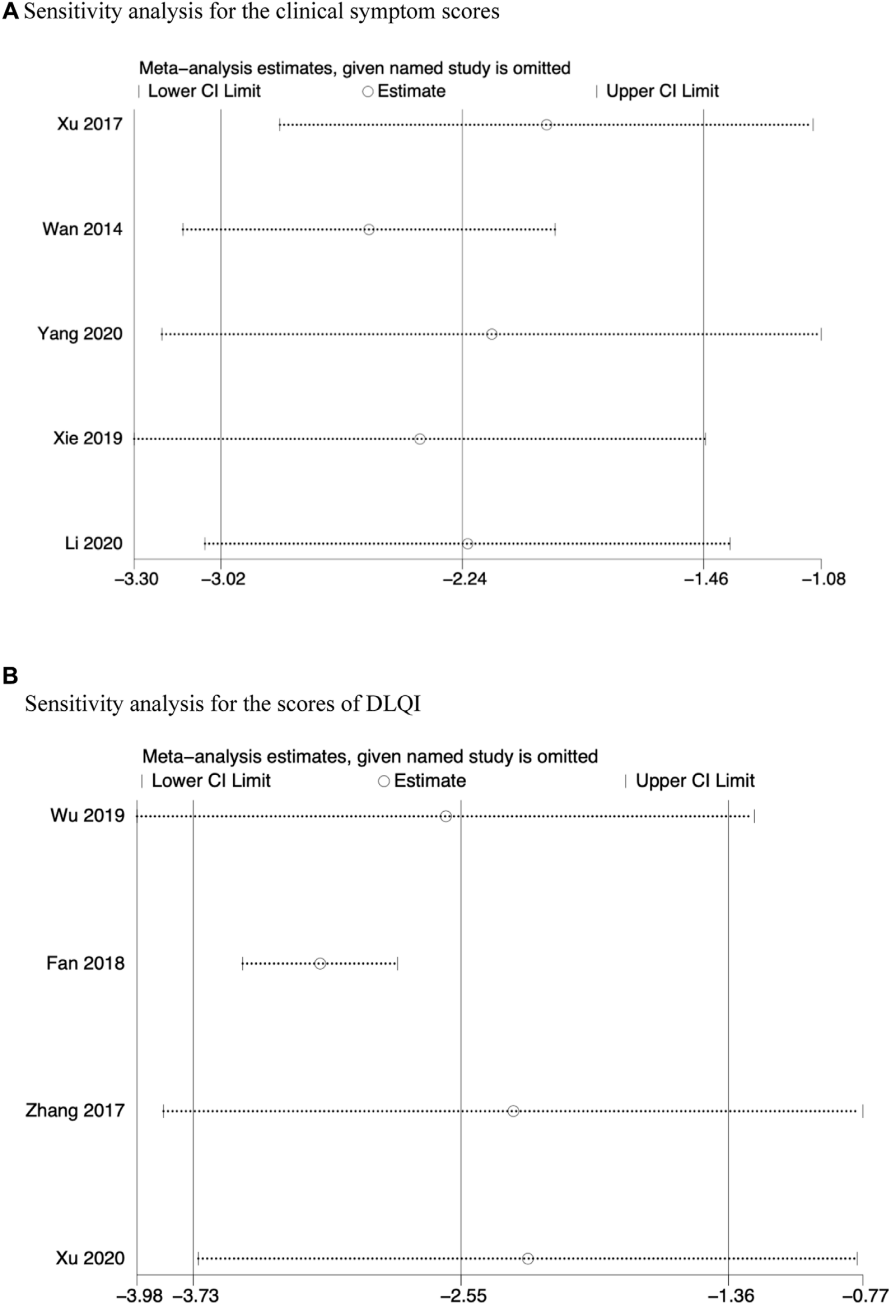
Sensitivity analysis for the clinical symptom scores.

We conducted a pooled analysis of all the five trials (Wan et al., 2017; Xu et al., 2019; Xie, 2020; Li, 2021; Yang, 2022) mentioned above and observed that the CHM and standard biomedical treatment combination therapy afforded a greater mean reduction in the clinical symptom score than that achieved using other treatment methods, with statistically significant between-study heterogeneity. (Weighted mean difference (WMD) = −2.24, 95%.CI [−3.02, −1.46], *p* < 0.00001, *I* ^2^ = 81.8%, random effects model; Supplementary Figure S1A). Therefore, we conducted a subgroup analysis (Table 2) and obtained significant differences irrespective of whether physical therapy (*p* < 0.00001, Figure 4A), topical drugs (*p* < 0.00001, Figure 4B) is used or not, and systemic drug types (*p* < 0.00001, Figure 4C). Further sensitivity analysis revealed no significant variations in the outcomes. Each result demonstrated strong agreement Figure 5A.

**TABLE 2:** Subgroup analysis for outcomes.

Subgroup	The clinical symptom scores				The scores of DLQI				The TCM syndrome scores			
	Study	WMD [95% CI]	*p*-value	*I* ^2^ (%)	Study	WMD [95% CI]	*p*-value	*I* ^2^ (%)	Study	WMD [95% CI]	*p*-value	*I* ^2^ (%)
Total	5	−2.24 (−3.02, −1.46)	<0.0001	81.8	4	−2.55 (−3.73, −1.36)	<0.0001	93.6	5	−4.42 (−5.33, −3.50)	<0.0001	92.1
whether physical therapy is used or not												
Yes (Group 1)	1	−2.46 (−4.71, −0.21)	NA	NA	1	−1.33 (−1.74, −0.92)	NA	NA	2	−5.65 (−6.42, −4.89)	0.504	0
No (Group 2)	4	−2.22 (−3.07, −1.37)	<0.0001	86.3	3	−3.17 (−3.51, −2.83)	0.451	0	3	−3.76 (−4.74, −2.78)	<0.0001	93.1
whether topical drug are used or not												
Yes (Group 1)	1	−2.46 (−4.71, −0.21)	NA	NA	3	−3.17 (−3.51, −2.83)	0.451	0	2	−3.70 (−5.62, −1.78)	<0.0001	92.2
No (Group 2)	4	−2.22 (−3.07, −1.37)	<0.0001	86.3	1	−1.33 (−1.74, −0.92)	NA	NA	3	−4.98 (−6.33, −3.62)	<0.0001	88.5
different systemic drug types												
Antibiotic (Group 1)	3	−2.11 (−3.35,−0.87)	0.006	80.4	2	−1.46 (−2.08, −0.83)	0.272	17.0	2	−4.60 (−6.85, −2.55)	<0.0001	95.2
Macrolides (Group 2)	0	NA	NA	NA	0	NA	NA	NA	0	NA	NA	NA
Immunosuppressor (Group 3)	1	−2.56 (−2.68, −2.44)	NA	NA	0	NA	NA	NA	0	NA	NA	NA
Not use (Group 4)	1	−2.46 (−4.71, −0.21)	NA	NA	2	−3.21 (−3.56, −2.86)	0.579	0	3	−4.20 (−4.85, −3.55)	0.149	52.0

NA, not available.

Weighted mean difference (WMD).

### 3.5 Impact of CHM on the score of DLQI

**FIGURE 6:**
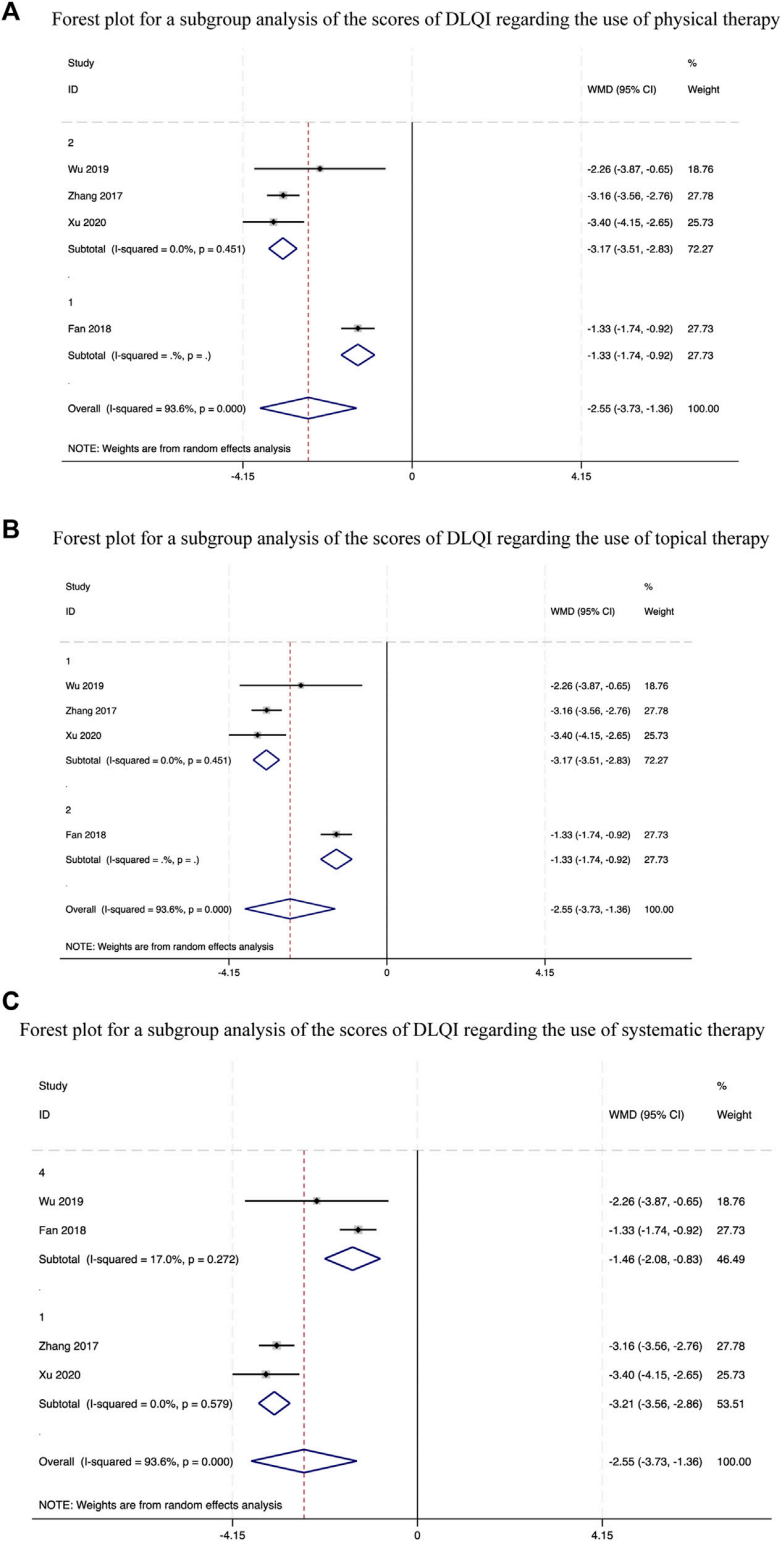
Forest plots for a subgroup analysis of the scores of DLQI.

A combined analysis of the four studies (Wu, 2020; Zhang et al., 2021b; Xu and Chen, 2022; Fan et al., 2023) that evaluated the scores of DLQI after the intervention revealed statistically significant between-study heterogeneity and a higher probability of score reduction when participants received the CHM and standard biomedical treatment combination therapy (WMD = −2.55, 95%.CI [−3.73, −1.36], *p* < 0.00001, *I* ^2^ = 93.6%, random effects model; Supplementary Figure S1B). We also conducted a subgroup analysis (Table 2) for this outcome measure. The analysis showed significant differences regardless of whether physical therapy (*p* < 0.00001, Figure 6A), topical drugs (*p* < 0.00001, Figure 6B) are used or not, and systemic drug types (*p* < 0.00001, Figure 6C). Further sensitivity analysis revealed no appreciable variations in the outcomes, and each result demonstrated strong agreement (Figure 5B).

### 3.6 Impact of CHM on TCM syndrome score

**FIGURE 7:**
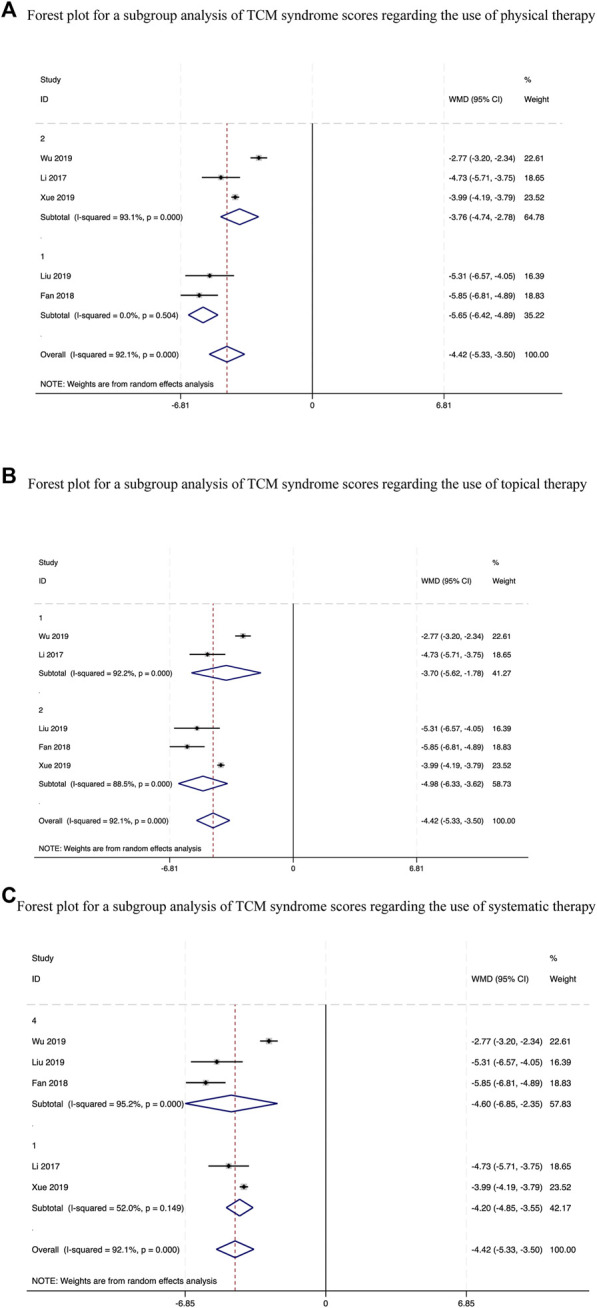
Forest plots for a subgroup analysis of TCM syndrome scores.

Five studies (Wu, 2020; Li et al., 2021; Yin, 2021; Fan et al., 2023; Liu et al., 2023) evaluated the weighted mean difference (WMD) of the TCM syndrome score during therapy. Combining the data obtained from these studies showed that the CHM and standard biomedical treatment combination therapy more effectively lowered the scores compared to those achieved using alternative placebo treatments, with statistically significant between-study heterogeneity (WMD = −4.42, 95%.CI [-5.33, −3.50], *p* < 0.00001, I^2^ = 92.1%, random effects model; Supplementary Figure S1C). Therefore, we conducted a subgroup analysis (Table 2) and noted significant differences irrespective of whether physical therapy (*p* < 0.00001, Figure 7A), topical drugs (*p* < 0.00001, Figure 7B) are used or not, and systemic drug types (*p* < 0.00001, Figure 7C).

### 3.7 Impact of CHM on EI

Three studies (Zhang et al., 2021b; Xu and Chen, 2022; Yang, 2022) evaluated post-treatment changes in the EI of the participants. Combining the data from these three studies revealed statistically significant between-study heterogeneity and a greater mean weight decrease with CHM and standard biomedical treatment combination treatment than that achieved with other treatments. (WMD = −151.65, 95%.CI [-276.06, −27.23], *p* < 0.00001, *I* ^2^ = 99.4%, random effects model; Supplementary Figure S1D).

### 3.8 Impact of CHM on IL-37

The WMD of the IL-37 level following treatment was evaluated in three trials (Li, 2021; Yin, 2021; Yang, 2022). Combining the data from these studies showed that the CHM and standard biomedical treatment combination therapy could more effectively lower the scores compared to that achieved with alternative placebo treatments. Additionally, no discernible between-study heterogeneity was noted (WMD = −4.23, 95%CI [−4.95, −3.51], *p* = 0.854, *I* ^2^ = 0%, random effects model; Supplementary Figure S1E).

### 3.9 Impact of CHM on the overall effective rate, recurrence rate, and adverse effects

#### 3.9.1 Overall effective rate

Thirteen trials (Wan et al., 2017; Xu et al., 2019; Mao and Wei, 2020; Wu, 2020; Xie, 2020; Li, 2021; Zhang et al., 2021b; Li et al., 2021; Yin, 2021; Xu and Chen, 2022; Yang, 2022; Fan et al., 2023; Liu et al., 2023) evaluated the relative risk of the overall effective rate during treatment. When the data from these studies were combined, no significant between-study heterogeneity was obtained. In addition, the overall efficiency rate was significantly lower with the CHM and standard biomedical treatment combination treatment than that achieved with other treatments (risk ratio (RR) = 1.25, 95%CI [1.18, 1.32], *p* = 0.994, *I* ^2^ = 0%, fixed effects model; Supplementary Figure S1F).

#### 3.9.2 Recurrence rate

Three trials (Mao and Wei, 2020; Xie, 2020; Li, 2021) evaluated the relative risk of the recurrence rate during therapy. When the data from these studies were combined, no significant between-study heterogeneity was observed. Moreover, the RR of the recurrence rate was significantly lower with the CHM and standard biomedical treatment combination therapy than that obtained using other therapies (RR = 0.27, 95%CI [0.15, 0.46], *p* = 0.297, I^2^ = 17.7%, fixed effects model; Supplementary Figure S1G).

#### 3.9.3 Adverse effects

Nine RCTs reported negative outcomes (Xu et al., 2019; Mao and Wei, 2020; Wu, 2020; Xie, 2020; Li, 2021; Zhang et al., 2021b; Xu and Chen, 2022; Yang, 2022; Fan et al., 2023). The groups receiving therapy and the control group were not much different from each other. The main side effects reported in these nine RCTs were gastrointestinal symptoms (such as nausea, vomiting, and diarrhea) and topical skin symptoms (such as redness, swelling, and itching). All adverse reactions resolved spontaneously or after symptomatic treatment. Only one case reported an adverse reaction associated with CHM induced by eating extremely cold food, which also resolved spontaneously (Li, 2021). In general, no significant adverse reactions (such as any injury to the liver and kidney or an abnormal blood routine) were noted.

### 3.10 Publication bias

**FIGURE 8:**
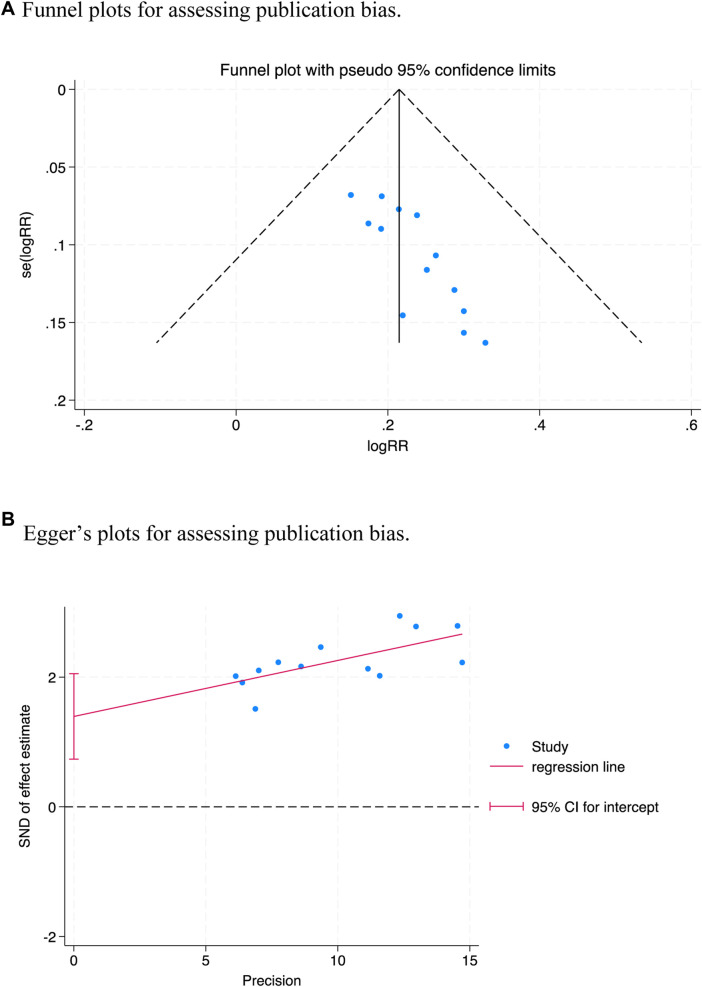
Plots for assessing publication bias.

The funnel plot (Figure 8A) showed a significant asymmetry. Egger’s test (Figure 8B) denoted a potential publication bias in the overall effective rate (*t* = 4.65, *p* = 0.001 < 0.05).

## 4 Discussion

Our findings demonstrate that, compared to conventional anti-rosacea therapies including medication such as metronidazole, clindamycin gels macrolides and antibiotics, surgery, physical therapy such as red and blue light irradiation, and laser therapy, and general treatment, the CHM and standard biomedical treatment combination therapy can afford better overall effective rates and lead to higher reductions in the TCM syndrome and clinical symptom scores, as well as in the scores of DLQI, EI, and IL-37 levels. Moreover, this combination leads to a lower recurrence rate compared to that observed with the basic Western treatment alone. Knowing the overall effective rate helps in choosing the most suitable treatment options for patients, improving the likelihood of positive outcomes and patient satisfaction. The recurrence rate helps in assessing the risk of disease recurrence or symptom relapse after an initial treatment course. This information is vital for determining the need for long-term follow-up or maintenance therapy. These findings support the usefulness of CHM and standard biomedical treatment combination therapy as a therapeutic approach that can improve rosacea management.

We utilized GRADEpro to evaluate the quality of the evidence (Table 3). The evaluation revealed that all evidence for the outcomes was of low quality, except for IL-37 and the overall effective rate, which had moderately high-quality evidence. The high degree of study heterogeneity and the poor methodological quality were the key reasons for the low certainty of the evidence. Hence, our study findings should be used with caution in clinical settings. More high-quality RCTs are required to assess the efficacy of our proposed therapeutic approach.

**TABLE 3:** Certainty of evidence.

Certainty assessment							No of patients		Effect		Certainty	Importance
No of studies	Study design	Risk of bias	Inconsistency	Indirectness	Imprecision	Other considerations	TCM	Control treatment	Relative (95% CI)	Absolute (95% CI)		
the clinical symptom score												
5	randomised trials	serious^a^	serious^b^	not serious	not serious	none	203	197	-	see comment	⊕⊕○○Low	IMPORTANT
the TCM syndrome scores												
5	randomised trials	serious^a^	serious^b^	not serious	not serious	none	224	224	-	see comment	⊕⊕○○Low	IMPORTANT
the scores of DLQI												
4	randomised trials	serious^a^	serious^b^	not serious	not serious	none	276	276	-	see comment	⊕⊕○○Low	IMPORTANT
EI												
3	randomised trials	serious^a^	serious^b^	not serious	not serious	none	226	226	-	see comment	⊕⊕○○Low	IMPORTANT
IL-37												
3	randomised trials	serious^a^	not serious	not serious	not serious	none	114	114	-	see comment	⊕⊕⊕○Moderate	IMPORTANT
The overall effective rate												
13	randomised trials	serious^a^	not serious	not serious	not serious	none	603/677 (89.1%)	479/671 (71.4%)	not pooled	see comment	⊕⊕⊕○Moderate	IMPORTANT

CI, confidence interval; MD, mean difference; RR, risk ratio.

Explanations.

a The risk of bias assessment is mostly “unclear risk” in articles.

b Here is serious heterogeneity among the studies included in the analysis of this outcome.

In the past, rosacea treatments included skin care and cosmetic treatments, topical therapies, oral therapies, laser and light-based therapies, injection therapies, treatments for specific types of rosacea, treatments for systemic comorbidities, and combination therapies. In recent years, biologics such as secukinumab, erenumab, and B244 topical spray are gaining increasing attention as new options for rosacea treatment. These therapeutic advances have increased treatment options and could improve the prognosis of patients with rosacea. Although the goal is to achieve complete or near-complete elimination of rosacea characteristics, all patients are currently unable to achieve these results despite adhering to the treatment protocol. Most studies were focused on using standard biomedical treatment for rosacea. However, the effectiveness of TCM and the integrated TCM and standard biomedical treatment therapy in treating rosacea cannot be ignored. As mentioned in this article, the TCM and the integrated TCM and standard biomedical treatment combination therapy, in particular, are more advantageous in terms of the overall effective rate and recurrence rate.

Current treatments almost exclusively target the two major features of rosacea: erythema and papules/pustules. Ocular rosacea and phyma have largely been neglected, even when medical interventions began in the early inflammatory stages (van Zuuren et al., 2021). To fully meet the needs of all patients with rosacea, further advances in physiopathology and treatment are needed.

According to conventional TCM, rosacea falls under the category of “facial sores” and is primarily brought on by heat-related bad depression in the meridian to the face, stomach, or large intestine (Chen et al., 2018). TCM frequently uses chrysanthemum, forsythia, honeysuckle, and scutellaria to treat rosacea (Wan et al., 2017; Xu et al., 2019; Mao and Wei, 2020; Wu, 2020; Xie, 2020; Li, 2021; Zhang et al., 2021b; Li et al., 2021; Yin, 2021; Heinrich et al., 2022; Xu and Chen, 2022; Yang, 2022; Fan et al., 2023; Liu et al., 2023). Research on animals has shown that honeysuckle can control the NF-κB signaling pathway in acne sufferers, which in turn can control the serum levels of IL-1β and TNF-α as well as the levels of intracellular NF-κB65, IKK-α, and IKK-β egg white Li et al., 2019. Forsythin, the active component of forsythia, has anti-inflammatory and wound-promoting properties (Chao et al., 2020). Baicalin and baicalin, the active ingredients in *Scutellaria baicalensis*, have antioxidant and anti-inflammatory properties (Zhu et al., 2020). Flavonoids, volatile oils, terpenoids, polysaccharides, and other compounds found in wild chrysanthemum can suppress local skin lesions and lessen the skin immunological inflammation (Li et al., 2021).

Our study has several limitations too. One is the lack of knowledge about the long-term durability of the treatment; the included trials had durations ranging from four to 16 weeks. Second, the overall methodological quality of the included research is low, which could result in an overestimation of efficacy due to the inadequate information supplied by the majority of publications and the defective study design. Hence, the results should be carefully interpreted. Third, the source of heterogeneity could not be fully identified in subgroup analysis. The various dosage forms (such as tablets, granules, pills, and decoctions) and the content of CHM utilized in interventions could have contributed to heterogeneity. Fourth, although we are detailed in this analysis mentioned the composition, dosage and usage of CHM, there may be differences due to the preparation process of CHM, so we need to caution with the results of the analysis.

## 5 Conclusion

Our study shows that a combination therapy comprising TCM and standard biomedical treatment is more effective in reducing clinical symptom and TCM syndrome scores, as well as the scores of the DLQI, EI, and IL-37 levels in patients with rosacea. The results also suggest that the TCM and standard biomedical treatment combination therapy can reduce the recurrence rate and improve the overall effectiveness. By describing and assessing the ongoing clinical studies for the treatment of rosacea by integrating TCM and standard biomedical treatment, this systematic review and meta-analysis closes a significant gap in the literature. Our work offers a foundation and a point of reference for the treatment of rosacea, although additional research is required to validate the efficacy of this treatment in clinical settings. It is important to note that the studies included in this analysis have certain limitations, and the evidence carries a degree of uncertainty. Therefore, there is a pressing need to perform further high-quality research to corroborate these findings and minimize potential biases. Such research endeavors will contribute to a more cautious interpretation of the results and provide a more robust basis for developing effective treatment strategies for rosacea. To bolster the validity and dependability of clinical evidence regarding the efficaciousness and safety of CHM in the treatment of rosacea, more extensive RCTs should be carried out using higher-quality methodologies. These investigations will produce more accurate and thorough data, strengthening the argument in favor of the therapeutic potential of CHM in the management of rosacea.

## Data Availability

The original contributions presented in the study are included in the article/Supplementary Material, further inquiries can be directed to the corresponding author.
