# Supplementary material for: Combination of traditional Chinese medicine and standard biomedical treatment for rosacea: a systematic review and a meta-analysis
Source: Front Pharmacol. 2024 Aug 8;15:1397141. doi: 10.3389/fphar.2024.1397141 (PMC11338883; doi:10.3389/fphar.2024.1397141)
Supplement: Supplementary file 2 [file Table1.docx]

Supplementary Material

# Supplementary Table S1 The composition of the prescriptions

| **Study** | **Chinese name** | **Species, concentration** | **Quality control reported? (Y/N)** | **Chemical analysis reported? (Y/N)** |
| --- | --- | --- | --- | --- |
| Mao  (2020) | gān cǎo,5g | *Glycyrrhiza uralensis* Fisch. ex DC.[Fabaceae: Glycyrrhizae radix et rhizoma],5g | N | N |
|  | shǔ yù,20g | *Dioscorea opposita* Thunb. [Dioscoreaceae: Dioscoreae rhizoma]，20g |  |  |
|  | yì yǐ rén,20g | *Coix lacryma-jobi* L.[Poaceae; Coicis semen],20g |  |  |
|  | chì xiǎo dòu,15g | *Adzuki beans* [Fabaceae: Phaseoli fructus],15g |  |  |
|  | hé huān huā,3g | *Albizia julibrissin* Durazz. [Fabaceae: Albiziae flos],3g |  |  |
|  | fú líng,15g | *Poria cocos* (Schw.) Wolf [Polyporaceae: Poriae sclerotium],15g |  |  |
|  | dì huáng,10g | *Rehmannia glutinosa* (Gaertn.) DC. [Scrophulariaceae: Rehmanniae radix],10g |  |  |
|  | chì sháo,10g | *Paeonia lactiflora* Pall. [Paeoniaceae: Paeoniae radix rubra],10g |  |  |
|  | yuè jì,3g | *Rosa rugosa* Thunb. [Rosaceae: Rosae rugosae flos],3g |  |  |
|  | kǔ shēn,12g | *Sophora flavescens* Aiton [Fabaceae: Sophorae flavescentis radix],12g |  |  |
|  | huáng qín,10g | *Scutellaria baicalensis* Georgi [Lamiaceae: Scutellariae radix],10g |  |  |
|  | jīn yín huā,15g | *Lonicera japonica* Thunb. [Caprifoliaceae: Lonicerae japonicae flos],15g |  |  |
|  | líng xiāo hūa,3g | *Platycodon grandiflorus* (Jacq.) A.DC. [Campanulaceae: Platycodi radix],3g |  |  |
|  | shān bái pí,15g | *Cortex Phellodendri* [Rutaceae: Phellodendri cortex],15g |  |  |
|  | pí pā yè,10g | *Folium Eriobotryae* [Rosaceae: Eriobotryae folium],10g |  |  |
|  | sāng bái pí,10g | *Morus alba L.* [Moraceae: Mori cortex],10g |  |  |
| Xu  (2019) | Shí gāo, 30g | *Gypsum fibrosum* [Gyprosum: Gypsum fibrosum], 30g | N | N |
|  | Zhī mǔ, 10g | *Anemarrhena asphodeloides* Bunge [Asparagaceae: Anemarrhenae rhizoma], 10g |  |  |
|  | Shēng dì, 30g | *Rehmannia glutinosa* (Gaertn.) DC. [Scrophulariaceae: Rehmanniae radix], 30g |  |  |
|  | Xuán shēn, 10g | *Scrophularia ningpoensis* Hemsl. [Scrophulariaceae: Scrophulariae radix], 10g |  |  |
|  | Chì sháo, 10g | *Paeonia lactiflora* Pall. [Paeoniaceae: Paeoniae radix rubra], 10g |  |  |
|  | Shēng má, 3g | *Cimicifuga foetida* L. [Ranunculaceae: Cimicifugae rhizoma], 3g |  |  |
|  | Niú bàng zǐ, 10g | *Arctium lappa* L. [Asteraceae: Arctii fructus], 10g |  |  |
|  | Jīng jiè, 10g | *Schizonepeta tenuifolia* Briq. [Lamiaceae: Schizonepetae herba], 10g |  |  |
|  | Fáng fēng, 10g | *Saposhnikovia divaricata* (Turcz.) Schischk. [Apiaceae: Saposhnikoviae radix], 10g |  |  |
|  | Bái máo gēn, 30g | *Imperata cylindrica* (L.) Beauv. [Poaceae: Imperatae rhizoma], 30g |  |  |
|  | Jīn yín huā, 15g | *Lonicera japonica* Thunb. [Caprifoliaceae: Lonicerae japonicae flos], 15g |  |  |
|  | Gān cǎo, 6g | *Glycyrrhiza uralensis* Fisch. ex DC. [Fabaceae: Glycyrrhizae radix et rhizoma], 6g |  |  |
| Wan  （2017） | Pí pá yè, 10g | *Folium Eriobotryae* [Rosaceae: Eriobotryae folium], 10g | N | N |
|  | Sāng bái pí, 15g | *Morus alba* L. [Moraceae: Mori cortex], 15g |  |  |
|  | Dì gǔ pí, 12g | *Cortex Dipsaci* [Dipsacaceae: Dipsaci cortex], 12g |  |  |
|  | Huáng qín, 15g | *Scutellaria baicalensis* Georgi [Lamiaceae: Scutellariae radix], 15g |  |  |
|  | Huáng lián, 6g | *Coptis chinensis* Franch. [Ranunculaceae: Coptidis rhizoma], 6g |  |  |
|  | Dān shēn, 15g | *Salvia miltiorrhiza* Bunge [Lamiaceae: Salviae miltiorrhizae radix], 15g |  |  |
|  | Méi guī huā, 15g | *Rosa rugosa* Thunb. [Rosaceae: Rosae rugosae flos], 15g |  |  |
|  | Líng xiāo huā, 15g | *Platycodon grandiflorus* (Jacq.) A.DC. [Campanulaceae: Platycodi radix], 15g |  |  |
|  | Jú huā, 20g | *Chrysanthemum indicum* L. [Asteraceae: Chrysanthemi flos], 20g |  |  |
|  | Hóng huā, 15g | *Carthamus tinctorius* L. [Asteraceae: Carthami flos], 15g |  |  |
|  | Chì sháo, 12g | *Paeonia lactiflora* Pall. [Paeoniaceae: Paeoniae radix rubra], 12g |  |  |
|  | Shēng dì, 20g | *Rehmannia glutinosa* (Gaertn.) DC. [Scrophulariaceae: Rehmanniae radix], 20g |  |  |
| Yang  （2022） | Sì jì qīng, 10g | *Elaeagnus pungens* Thunb. [Elaeagnaceae: Elaeagni folium], 10g | N | N |
|  | Nǚ zhēn zǐ, 15g | *Ligustrum lucidum* W.T. Aiton [Oleaceae: Ligustri lucidi fructus], 15g |  |  |
|  | Zhī mǔ, 15g | *Anemarrhena asphodeloides* Bunge [Asparagaceae: Anemarrhenae rhizoma], 15g |  |  |
|  | Lián qiào, 15g | *Forsythia suspensa* (Thunb.) Vahl [Oleaceae: Forsythiae fructus], 15g |  |  |
|  | Pí pá yè, 15g | *Folium Eriobotryae* [Rosaceae: Eriobotryae folium], 15g |  |  |
|  | Shā jí, 5g | *Hippophae rhamnoides* L. [Elaeagnaceae: Hippophae fructus], 5g |  |  |
| Xie  （2020） | Shēng shí gāo, 30g | *Gypsum fibrosum* [Gyprosum: Gypsum fibrosum], 30g | N | N |
|  | Zhī mǔ, 18g | *Anemarrhena asphodeloides* Bunge [Asparagaceae: Anemarrhenae rhizoma], 18g |  |  |
|  | Gān cǎo, 6g | *Glycyrrhiza uralensis* Fisch. ex DC. [Fabaceae: Glycyrrhizae radix et rhizoma], 6g |  |  |
|  | Jīng mǐ, 9g | *Oryza sativa* L. [Poaceae: Oryzae semen], 9g |  |  |
|  | Pú gōng yīng, 10g | *Taraxacum mongolicum* Hand.-Mazz. [Asteraceae: Taraxaci herba], 10g |  |  |
|  | Hóng huā, 10g | *Carthamus tinctorius* L. [Asteraceae: Carthami flos], 10g |  |  |
|  | Bái sháo, 10g | *Paeonia lactiflora* Pall. [Paeoniaceae: Paeoniae radix alba], 10g |  |  |
|  | Jiàn qū, 10g | *Curcuma zedoaria* (Christm.) Roscoe [Zingiberaceae: Zedoariae rhizoma], 10g |  |  |
| Li  （2021） | Huáng bò, 15g | *Phellodendron chinense* C.K.Schneid. [Rutaceae: Phellodendri cortex], 15g | N | N |
|  | Shā rén, 10g | *Amomum villosum* Lour. [Zingiberaceae: Amomi fructus rotundus], 10g |  |  |
|  | Gān cǎo, 10g | *Glycyrrhiza uralensis* Fisch. ex DC. [Fabaceae: Glycyrrhizae radix et rhizoma], 10g |  |  |
| Wu  （2020） | Qīng bàn xià, 9g | *Pinellia ternata* (Thunb.) Makino [Araceae: Pinelliae rhizoma praeparatum], 9g | N | N |
|  | Wǔ wèi zǐ, 9g | *Schisandra chinensis* (Turcz.) Baill. [Schisandraceae: Schisandrae fructus], 9g |  |  |
|  | Zhǐ shí, 9g | *Aurantium fructus immaturus* [Rutaceae: Citri unripe fructus], 9g |  |  |
|  | Fú líng, 9g | *Poria cocos* (Schw.) Wolf [Polyporaceae: Poria], 9g |  |  |
|  | Hé zǐ, 9g | *Terminalia chebula* Retz. [Combretaceae: Chebulae fructus], 9g |  |  |
|  | Gān jiāng, 9g | *Zingiber officinale* Roscoe [Zingiberaceae: Zingiberis rhizoma], 9g |  |  |
|  | Chén pí, 9g | *Citrus reticulata* Blanco [Rutaceae: Citri reticulatae pericarpium], 9g |  |  |
|  | Shēng suān zǎo rén, 30g | *Ziziphus jujuba* Mill. var. spinosa (Bunge) Hu ex H.F.Chou [Rhamnaceae: Zizyphi spinosae semen], 30g |  |  |
|  | Chǎo gān cǎo, 9g | *Glycyrrhiza uralensis* Fisch. ex DC. [Fabaceae: Glycyrrhizae radix et rhizoma], 9g |  |  |
|  | Dà zǎo | *Ziziphus jujuba* Mill. [Rhamnaceae: Zizyphi fructus], 按需使用 |  |  |
| Liu  （2023） | Zhú yè, 15g | *Bambusae folium*, 15g | N | N |
|  | Chái hú, 15g | *Bupleurum chinense* DC. [Apiaceae: Bupleuri radix], 15g |  |  |
|  | Bái sháo, 15g | *Paeonia lactiflora* Pall. [Paeoniaceae: Paeoniae radix alba], 15g |  |  |
|  | Lián qiào, 10g | *Forsythia suspensa* (Thunb.) Vahl [Oleaceae: Forsythiae fructus], 10g |  |  |
|  | Mǔ dān pí, 10g | *Paeonia suffruticosa* Andrews [Paeoniaceae: Moutan cortex], 10g |  |  |
|  | Mǔ lì, 20g | *Ostrea gigas* Thunberg [Ostreidae: Ostreae concha], 20g |  |  |
|  | Lóng gǔ, 20g | *Draconis ossis*, 20g |  |  |
|  | Rěn dōng téng, 20g | *Lonicera japonica* Thunb. [Caprifoliaceae: Lonicerae japonicae caulis], 20g |  |  |
|  | Zǐ jìng pí, 15g | *Cercis chinensis* Bunge [Fabaceae: Cercidis chinensis cortex], 15g |  |  |
|  | Chuān shè gān, 6g | *Erodium stephanianum* Willd. [Geraniaceae: Erodii herba], 6g |  |  |
|  | Bái huā shé shé cǎo, 15g | *Hedyotis diffusa* Willd. [Rubiaceae: Hedyotidis diffusae herba], 15g |  |  |
|  | Pú gōng yīng, 15g | *Taraxacum mongolicum* Hand.-Mazz. [Asteraceae: Taraxaci herba], 15g |  |  |
|  | Yì yǐ rén, 20g | *Coix lacryma-jobi* L. var. ma-yuen (Rom.Caill.) Stapf [Poaceae: Coicis semen], 20g |  |  |
|  | Zhòng lóu, 10g | *Stellera chamaejasme* L. [Thymelaeaceae: Stellerae radix], 10g |  |  |
|  | Bài jiàng cǎo, 10g | *Astragalus membranaceus* (Fisch.) Bunge [Fabaceae: Astragali radix], 10g |  |  |
|  | Hàn mò lián, 15g | *Erodium cicutarium* (L.) L'Her. ex Aiton [Geraniaceae: Erodii herba], 15g |  |  |
|  | Nǚ zhēn zǐ, 30g | *Ligustrum lucidum* W.T. Aiton [Oleaceae: Ligustri lucidi fructus], 30g |  |  |
|  | Gān cǎo, 3g | *Glycyrrhiza uralensis* Fisch. ex DC. [Fabaceae: Glycyrrhizae radix et rhizoma], 3g |  |  |
| Fan  （2023） | Lóng dǎn cǎo, 9g | *Gentiana scabra* Bunge [Gentianaceae: Gentianae scabrae herba], 9g | N | N |
|  | Máo gēn, 30g | *Imperata cylindrica* (L.) Beauv. [Poaceae: Imperatae rhizoma], 30g |  |  |
|  | Shēng dì huáng, 15g | *Rehmannia glutinosa* (Gaertn.) DC. [Scrophulariaceae: Rehmanniae radix], 15g |  |  |
|  | Dà qīng yè, 15g | *Herba Isatidis,* 15g |  |  |
|  | Chē qián cǎo, 15g | *Plantago asiatica* L. [Plantaginaceae: Plantaginis herba], 15g |  |  |
|  | Shēng shí gāo, 30g | *Gypsum fibrosum* [Gyprosum: Gypsum fibrosum], 30g |  |  |
|  | Huáng qín, 9g | *Scutellaria baicalensis* Georgi [Lamiaceae: Scutellariae radix], 9g |  |  |
|  | Huá shí, 15g | *Talcum* [Talcum], 15g |  |  |
|  | Fú líng, 10g | *Poria cocos* (Schw.) Wolf [Polyporaceae: Poria], 10g |  |  |
|  | Màn jīng zǐ, 8g | *Vitex negundo* L. [Lamiaceae: Viticis fructus], 8g |  |  |
|  | Gān cǎo, 15g | *Glycyrrhiza uralensis* Fisch. ex DC. [Fabaceae: Glycyrrhizae radix et rhizoma], 15g |  |  |
|  | Yì yǐ rén, 6g | *Coix lacryma-jobi* L. var. ma-yuen (Rom.Caill.) Stapf [Poaceae: Coicis semen], 6g |  |  |
|  | Jīng jiè, 12g | *Schizonepeta tenuifolia* Briq. [Lamiaceae: Schizonepetae herba], 12g |  |  |
| Li  （2021） | Nán shā shēn, 15g | *Panax notoginseng* (Burkill) F.H.Chen [Araliaceae: Panacis notoginseng radix], 15g |  |  |
|  | Fú líng, 15g | *Poria cocos* (Schw.) Wolf [Polyporaceae: Poria], 15g |  |  |
|  | Chǎo bái zhú, 15g | *Atractylodes macrocephala* Koidz. [Asteraceae: Atractylodis macrocephalae rhizoma], 15g |  |  |
|  | Shēng dì huáng, 15g | *Rehmannia glutinosa* (Gaertn.) DC. [Scrophulariaceae: Rehmanniae radix], 15g |  |  |
|  | Jīn yín huā, 12g | *Lonicera japonica* Thunb. [Caprifoliaceae: Lonicerae japonicae flos], 12g |  |  |
|  | Lián qiào, 12g | *Forsythia suspensa* (Thunb.) Vahl [Oleaceae: Forsythiae fructus], 12g |  |  |
|  | Lú gēn, 12g | *Phragmites australis* (Cav.) Trin. ex Steud. [Poaceae: Phragmitis rhizoma], 12g |  |  |
|  | Qīng hāo, 12g | *Artemisia annua* L. [Asteraceae: Artemisiae annuae herba], 12g |  |  |
|  | Dān shēn, 12g | *Salvia miltiorrhiza* Bunge [Lamiaceae: Salviae miltiorrhizae radix], 12g |  |  |
|  | Sāng yè, 9g | *Morus alba* L. [Moraceae: Mori folium], 9g |  |  |
|  | Chì sháo, 9g | *Paeonia lactiflora* Pall. [Paeoniaceae: Paeoniae radix rubra], 9g |  |  |
|  | Yě jú huā, 9g | *Aster tataricus* L.f. [Asteraceae: Asteris tatarici flos], 9g |  |  |
|  | Jiāo zhī zǐ, 6g | *Gardenia jasminoides* Ellis [Rubiaceae: Gardeniae fructus], 6g |  |  |
|  | Dān pí, 6g | *Paeonia suffruticosa* Andrews [Paeoniaceae: Moutan cortex], 6g |  |  |
|  | Huáng qín, 6g | *Scutellaria baicalensis* Georgi [Lamiaceae: Scutellariae radix], 6g |  |  |
| Zhang  （2021） | Pí pá yè, 15g | *Folium Eriobotryae* [Rosaceae: Eriobotryae folium], 15g |  |  |
|  | Sāng bái pí, 15g | *Morus alba* L. [Moraceae: Mori cortex], 15g |  |  |
|  | Chì sháo, 15g | *Paeonia lactiflora* Pall. [Paeoniaceae: Paeoniae radix rubra], 15g |  |  |
|  | Zhī mǔ, 15g | *Anemarrhena asphodeloides* Bunge [Asparagaceae: Anemarrhenae rhizoma], 15g |  |  |
|  | Jīn yín huā, 15g | *Lonicera japonica* Thunb. [Caprifoliaceae: Lonicerae japonicae flos], 15g |  |  |
|  | Bái huā shé shé cǎo, 15g | *Hedyotis diffusa* Willd. [Rubiaceae: Hedyotidis diffusae herba], 15g |  |  |
|  | Mǔ dān pí, 15g | *Paeonia suffruticosa* Andrews [Paeoniaceae: Moutan cortex], 15g |  |  |
|  | Bái máo gēn, 15g | *Imperata cylindrica* (L.) Beauv. [Poaceae: Imperatae rhizoma], 15g |  |  |
|  | Xuán shēn, 20g | *Scrophularia ningpoensis* Hemsl. [Scrophulariaceae: Scrophulariae radix], 20g |  |  |
|  | Dì huáng, 20g | *Rehmannia glutinosa* (Gaertn.) DC. [Scrophulariaceae: Rehmanniae radix], 20g |  |  |
|  | Huáng qín, 20g | *Scutellaria baicalensis* Georgi [Lamiaceae: Scutellariae radix], 20g |  |  |
|  | Chén pí, 20g | *Citrus reticulata* Blanco [Rutaceae: Citri reticulatae pericarpium], 20g |  |  |
|  | Gān cǎo, 10g | *Glycyrrhiza uralensis* Fisch. ex DC. [Fabaceae: Glycyrrhizae radix et rhizoma], 10g |  |  |
| Xu  （2022） | Shēng shí gāo, 30g | *Gypsum fibrosum* [Gyprosum: Gypsum fibrosum], 30g | N | N |
|  | Zhī mǔ, 10g | *Anemarrhena asphodeloides* Bunge [Asparagaceae: Anemarrhenae rhizoma], 10g |  |  |
|  | Jīn yín huā, 12g | *Lonicera japonica* Thunb. [Caprifoliaceae: Lonicerae japonicae flos], 12g |  |  |
|  | Lián qiào, 12g | *Forsythia suspensa* (Thunb.) Vahl [Oleaceae: Forsythiae fructus], 12g |  |  |
|  | Zǐ huā dì dīng, 12g | *Hedyotis diffusa* Willd. [Rubiaceae: Hedyotidis diffusae herba], 12g |  |  |
|  | Pú gōng yīng, 12g | *Taraxacum mongolicum* Hand.-Mazz. [Asteraceae: Taraxaci herba], 12g |  |  |
|  | Pí pá yè, 15g | *Folium Eriobotryae* [Rosaceae: Eriobotryae folium], 15g |  |  |
|  | Huáng qín, 10g | *Scutellaria baicalensis* Georgi [Lamiaceae: Scutellariae radix], 10g |  |  |
|  | Xià kū cǎo, 15g | *Prunella vulgaris* L. [Lamiaceae: Prunellae spica], 15g |  |  |
|  | Shēng dì, 15g | *Rehmannia glutinosa* (Gaertn.) DC. [Scrophulariaceae: Rehmanniae radix], 15g |  |  |
|  | Chì sháo, 10g | *Paeonia lactiflora* Pall. [Paeoniaceae: Paeoniae radix rubra], 10g |  |  |
|  | Mǔ dān pí, 10g | *Paeonia suffruticosa* Andrews [Paeoniaceae: Moutan cortex], 10g |  |  |
|  | Hǎi zǎo, 10g | *Seaweed* [Algae: Sargassum], 10g |  |  |
|  | Kūn bù, 10g | *Laminaria japonica* Aresch. [Laminariaceae: Laminariae thallus], 10g |  |  |
|  | Māo zhuā cǎo, 10g | *Uncaria rhynchophylla* (Miq.) Miq. ex Havil. [Rubiaceae: Uncariae ramulus cum uncis], 10g |  |  |
| Xue  （2021） | Huáng qín, 10g | *Scutellaria baicalensis* Georgi [Lamiaceae: Scutellariae radix], 10g | N | N |
|  | Lián qiào, 10g | *Forsythia suspensa* (Thunb.) Vahl [Oleaceae: Forsythiae fructus], 10g |  |  |
|  | Tiān huā fěn, 10g | *Trichosanthes kirilowii* Maxim. [Cucurbitaceae: Trichosanthis semen], 10g |  |  |
|  | Gé gēn, 10g | *Pueraria lobata* (Willd.) Ohwi [Fabaceae: Puerariae radix], 10g |  |  |
|  | Fáng fēng, 6g | *Saposhnikovia divaricata* (Turcz.) Schischk. [Apiaceae: Saposhnikoviae radix], 6g |  |  |
|  | Chuān xiōng, 10g | *Ligusticum chuanxiong* Hort. [Apiaceae: Chuanxiong rhizoma], 10g |  |  |
|  | Dāng guī, 10g | *Angelica sinensis* (Oliv.) Diels [Apiaceae: Angelicae sinensis radix], 10g |  |  |
|  | Chì sháo, 10g | *Paeonia lactiflora* Pall. [Paeoniaceae: Paeoniae radix rubra], 10g |  |  |
|  | Shēng dì huáng, 10g | *Rehmannia glutinosa* (Gaertn.) DC. [Scrophulariaceae: Rehmanniae radix], 10g |  |  |
|  | Shēng gān cǎo, 10g | *Glycyrrhiza uralensis* Fisch. ex DC. [Fabaceae: Glycyrrhizae radix et rhizoma], 10g |  |  |

# Supplementary Table S2 The detailed compositions of CHM

| Study | Chinese Herbal Medicine | ﻿Ingredients of herb prescription | | Usage |
| --- | --- | --- | --- | --- |
|  |  | Latin name | Chinses name |  |
| Mao  (2020)^[10]^ | Rose Yurong decoction | *Glycyrrhiza uralensis* Fisch,5g  *Dioscorea opposita* Thunb,20g  *Coix lacryma-jobi* L,20g  *Vigna umbellata* (Thunb.) Ohwi & Ohashi,15g  *Albizia julibrissin* Durazz,3g  *Poria cocos* (Schw.) Wolf,15g  *Rehmannia glutinosa* (Gaertn.) DC,10g  *Paeonia lactiflora* Pall,10g  *Rosa rugosa* Thunb,10g  *Sophora flavescens* Aiton,12g  *Scutellaria baicalensis* Georgi,10g  *Lonicera japonica* Thunb,15g  *Platycodon grandiflorus* (Jacq.) A.DC,3g  *Cortex Phellodendri,15g*  *Folium Eriobotryae,10g*  *Morus alba L,10g* | gān cǎo,5g  shǔ yù,20g  yì yǐ rén,20g  chì xiǎo dòu,15g  hé huān huā,3g  fú líng,15g  dì huáng,10g  chì sháo,10g  yuè jì,3g  kǔ shēn,12g  huáng qín,10g  jīn yín huā,15g  líng xiāo hūa,3g  shān bái pí,15g  pí pā yè,10g  sāng bái pí,10g | ﻿1 package bid |
| Xu (2019)^[11]^ | Liangxue Xiaofeng Powder | *Gypsum fibrosum,30g*  *Anemarrhena asphodeloides* Bunge,10g  *Rehmannia glutinosa* (Gaertn.) DC,30g  *Scrophularia ningpoensis* Hemsl,10g  *Paeonia lactiflora* Pall,10g  *Cimicifuga foetida* L,3g  *Arctium lappa* L,10g  *Schizonepeta tenuifolia* Briq,10g  *Saposhnikovia divaricata* (Turcz.) Schischk,10g  *Imperata cylindrica* (L.) Beauv,10g  *Lonicera japonica* Thunb,15g  *Glycyrrhiza uralensis* Fisch,6g | Shí gāo, 30g  Zhī mǔ, 10g  Shēng dì, 30g  Xuán shēn, 10g  Chì sháo, 10g  Shēng má, 3g  Niú bàng zǐ, 10g  Jīng jiè, 10g  Fáng fēng, 10g  Bái máo gēn, 30g  Jīn yín huā, 15g  Gān cǎo, 6g | ﻿1 package bid |
| Wan (2017)^[12]^ | Liangxue Qingfei Powder | *Folium Eriobotryae,10g*  *Morus alba* L,15g  *Cortex Dipsaci,12g*  *Scutellaria baicalensis* Georgi,15g  *Coptis chinensis* Franch,15g  *Salvia miltiorrhiza* Bunge,15g  *Rosa rugosa* Thunb,15g  *Campsis grandiflora* (Thunb.)Schum,15g  *Chrysanthemum indicum* L,20g  *Carthamus tinctorius* L,15g  *Paeonia lactiflora* Pall,12g  *Rehmannia glutinosa* (Gaertn.) DC,20g | Pí pá yè, 10g  Sāng bái pí, 15g  Dì gǔ pí, 12g  Huáng qín, 15g  Huáng lián, 6g  Dān shēn, 15g  Méi guī huā, 15g  Líng xiāo huā, 15g  Jú huā, 20g  Hóng huā, 15g  Chì sháo, 12g  Shēng dì, 20g | 1 package bid |
| Yang (2022)^[13]^ | Qingfei Liangxue Decoction | *Elaeagnus pungens* Thunb,10g  *Ligustrum lucidum* W.T. Aiton,15g  *Anemarrhena asphodeloides* Bunge,15g  *Forsythia suspensa* (Thunb.) Vahl,15g  *Folium Eriobotryae,15g*  *Hippophae rhamnoides* L,5g | Sì jì qīng, 10g  Nǚ zhēn zǐ, 15g  Zhī mǔ, 15g  Lián qiào, 15g  Pí pá yè, 15g  Shā jí, 5g | 1 package bid |
| Xie (2020)^[14]^ | Jiawei Baihu Decoction | *Gypsum fibrosum,30g*  *Anemarrhena asphodeloides* Bunge,18g  *Glycyrrhiza uralensis* Fisch,6g  *Oryza sativa* L,9g  *Taraxacum mongolicum* Hand.-Mazz,10g  *Carthamus tinctorius* L,10g  *Paeonia lactiflora* Pall,10g  *Curcuma zedoaria* (Christm.) Roscoe,10g | Shēng shí gāo, 30g  Zhī mǔ, 18g  Gān cǎo, 6g  Jīng mǐ, 9g  Pú gōng yīng, 10g  Hóng huā, 10g  Bái sháo, 10g  Jiàn qū, 10g | 1 package bid |
| Li (2021)^[15]^ | Fengsui Pellet | *Phellodendron chinense* C.K.Schneid,15g  *Amomum villosum* Lour,10g  *Glycyrrhiza uralensis* Fisch,10g | Huáng bò, 15g  Shā rén, 10g  Gān cǎo, 10g | 1 package bid |
| Wu (2020)^[16]^ | Fuhe Decoction | *Pinellia ternata* (Thunb.) Makino,9g  *Schisandra chinensis* (Turcz.) Baill,9g  *Aurantium fructus immaturus,9g*  *Poria cocos* (Schw.) Wolf,9g  *Terminalia chebula* Retz,9g  *Zingiber officinale* Roscoe,9g  *Citrus reticulata* Blanco,9g  *Ziziphus jujuba* Mill. var. spinosa (Bunge) Hu ex H.F.Chou,30g  *Glycyrrhiza uralensis,9g*  *Ziziphus jujuba* Mill, 2 pills | Qīng bàn xià, 9g  Wǔ wèi zǐ, 9g  Zhǐ shí, 9g  Fú líng, 9g  Hé zǐ, 9g  Gān jiāng, 9g  Chén pí, 9g  Shēng suān zǎo rén, 30g  Chǎo gān cǎo, 9g  Dà zǎo，2 pills | 1 package bid |
| Liu (2023)^[18]^ | Chaishao Longmu Decoction | *Bambusae folium,15g*  *Bupleurum chinense* DC,15g  *Paeonia lactiflora* Pall,15g  *Forsythia suspensa* (Thunb.) Vahl,10g  *Paeonia suffruticosa* Andrews,10g  *Ostrea gigas* Thunberg,20g  *Draconis ossis,20g*  *Lonicera japonica* Thunb,20g  *Cercis chinensis* Bunge,15g  *Erodium stephanianum* Willd,6g  *Hedyotis diffusa* Willd,15g  *Taraxacum mongolicum* Hand.-Mazz,15g  *Coix lacryma-jobi* L,20g  *Stellera chamaejasme* L,10g  *Patrinia scabiosaefolia，10g*  *Erodium cicutarium* (L.) L'Her. ex Aiton,15g  *Ligustrum lucidum* W.T. Aiton,30g  *Glycyrrhiza uralensis* Fisch,3g | Zhú yè, 15g  Chái hú, 15g  Bái sháo, 15g  Lián qiào, 10g  Mǔ dān pí, 10g  Mǔ lì, 20g  Lóng gǔ, 20g  Rěn dōng téng, 20g  Zǐ jìng pí, 15g  Chuān shè gān, 6g  Bái huā shé shé cǎo, 15g  Pú gōng yīng, 15g  Yì yǐ rén, 20g  Zhòng lóu, 10g  Bài jiàng cǎo, 10g  Hàn mò lián, 15g  Nǚ zhēn zǐ, 30g  Gān cǎo, 3g | 1 package bid |
| Fan (2023)^[19]^ | Qingre Chushi Decoction | *Gentiana scabra* Bunge,9g  *Imperata cylindrica* (L.) Beauv,30g  *Rehmannia glutinosa* (Gaertn.) DC,15g  *Herba Isatidis,15g*  *Plantago asiatica* L,15g  *Gypsum fibrosum,30g*  *Scutellaria baicalensis* Georgi,30g  *Talcum,15g*  *Poria cocos* (Schw.) Wolf,10g  *Vitex negundo* L,8g  *Glycyrrhiza uralensis* Fisch,15g  *Coix lacryma-jobi* L,6g  *Schizonepeta tenuifolia* Briq,12g | Lóng dǎn cǎo, 9g  Máo gēn, 30g  Shēng dì huáng, 15g  Dà qīng yè, 15g  Chē qián cǎo, 15g  Shēng shí gāo, 30g  Huáng qín, 9g  Huá shí, 15g  Fú líng, 10g  Màn jīng zǐ, 8g  Gān cǎo, 15g  Yì yǐ rén, 6g  Jīng jiè, 12g | 1 package bid |
| Li (2021)^[20]^ | Yiqi Yangyin Sanxie Decoction | Adenophora tetraphylla*(Thunb.)*Fisch,15g  *Poria cocos* (Schw.) Wolf,15g  *Atractylodes macrocephala* Koidz,15g  *Rehmannia glutinosa* (Gaertn.) DC,15g  *Lonicera japonica* Thunb,12g  *Forsythia suspensa* (Thunb.) Vahl,12g  *Phragmites australis* (Cav.) Trin. ex Steud,12g  *Artemisia annua* L,12g  *Salvia miltiorrhiza* Bunge,12g  *Morus alba* L,9g  *Paeonia lactiflora* Pall,9g  *Aster tataricus* L.f,9g  *Gardenia jasminoides* Ellis,6g  *Paeonia suffruticosa* Andrews,6g  *Scutellaria baicalensis* Georgi,6g | Nán shā shēn, 15g  Fú líng, 15g  Chǎo bái zhú, 15g  Shēng dì huáng, 15g  Jīn yín huā, 12g  Lián qiào, 12g  Lú gēn, 12g  Qīng hāo, 12g  Dān shēn, 12g  Sāng yè, 9g  Chì sháo, 9g  Yě jú huā, 9g  Jiāo zhī zǐ, 6g  Dān pí, 6g  Huáng qín, 6g | 1 package bid |
| Zhang (2021)^[21]^ | LIangxue Qingfei Decoction | *Folium Eriobotryae,15g*  *Morus alba* L,15g  *Paeonia lactiflora* Pall,15g  *Anemarrhena asphodeloides* Bunge,15g  *Lonicera japonica* Thunb,15g  *Hedyotis diffusa* Willd,15g  *Paeonia suffruticosa* Andrews,15g  *Imperata cylindrica* (L.) Beauv,15g  *Scrophularia ningpoensis* Hemsl,20g  *Rehmannia glutinosa* (Gaertn.) DC,20g  *Scutellaria baicalensis* Georgi,20g  *Citrus reticulata* Blanco,20g  *Glycyrrhiza uralensis* Fisch,10g | Pí pá yè, 15g  Sāng bái pí, 15g  Chì sháo, 15g  Zhī mǔ, 15g  Jīn yín huā, 15g  Bái huā shé shé cǎo, 15g  Mǔ dān pí, 15g  Bái máo gēn, 15g  Xuán shēn, 20g  Dì huáng, 20g  Huáng qín, 20g  Chén pí, 20g  Gān cǎo, 10g | 1 package bid |
| Xu (2022)^[22]^ | Liangxue Qingfei Decoction | *Gypsum fibrosum,30g*  *Anemarrhena asphodeloides* Bunge,10g  *Lonicera japonica* Thunb,12g  *Forsythia suspensa* (Thunb.) Vahl,12g  *Hedyotis diffusa* Willd,12g  *Taraxacum mongolicum* Hand.-Mazz,12g  *Folium Eriobotryae,15g*  *Scutellaria baicalensis* Georgi,10g  *Prunella vulgaris* L,15g  *Rehmannia glutinosa* (Gaertn.) DC,15g  *Paeonia lactiflora* Pall,10g  *Paeonia suffruticosa* Andrews,10g  *Seaweed,10g*  *Laminaria japonica* Aresch,10g  *Uncaria rhynchophylla* (Miq.) Miq. ex Havil,10g | Shēng shí gāo, 30g  Zhī mǔ, 10g  Jīn yín huā, 12g  Lián qiào, 12g  Zǐ huā dì dīng, 12g  Pú gōng yīng, 12g  Pí pá yè, 15g  Huáng qín, 10g  Xià kū cǎo, 15g  Shēng dì, 15g  Chì sháo, 10g  Mǔ dān pí, 10g  Hǎi zǎo, 10g  Kūn bù, 10g  Māo zhuā cǎo, 10g | 1 package bid |
| Xue (2021)^[23]^ | Huangqin Qingfei Decoction | *Scutellaria baicalensis* Georgi,10g  *Forsythia suspensa* (Thunb.) Vahl,10g  *Trichosanthes kirilowii* Maxim,10g  *Pueraria lobata* (Willd.) Ohwi,10g  *Saposhnikovia divaricata* (Turcz.) Schischk,6g  *Ligusticum chuanxiong* Hort,10g  *Angelica sinensis* (Oliv.) Diels,10g  *Paeonia lactiflora* Pall,10g  *Rehmannia glutinosa* (Gaertn.) DC,10g  *Glycyrrhiza uralensis* Fisch,10g | Huáng qín, 10g  Lián qiào, 10g  Tiān huā fěn, 10g  Gé gēn, 10g  Fáng fēng, 6g  Chuān xiōng, 10g  Dāng guī, 10g  Chì sháo, 10g  Shēng dì huáng, 10g  Shēng gān cǎo, 10g | 1 package bid |

# 3 Supplementary Figure S1 Forest plots

a. the clinical symptom scores

b. the scores of DLQI

c. TCM syndrome scores

d. EI

e.IL-37

f. the overall effective rate

g. the recurrence rate

DLQI: Dermatology Life Quality Index; EI: erythema index; IL-37: interleukin 37 level

# 4 Supplementary Material Table S3 The PRISMA checklist of this meta-analysis

| **Section and Topic** | **Item #** | **Checklist item** | **Location**  **where item**  **is reported** |
| --- | --- | --- | --- |
| **TITLE** | | |  |
| Title | 1 | Identify the report as a systematic review. |  |
| **ABSTRACT** | | |  |
| Abstract | 2 | See the PRISMA 2020 for Abstracts checklist. |  |
| **INTRODUCTION** | | |  |
| Rationale | 3 | Describe the rationale for the review in the context of existing knowledge. |  |
| Objectives | 4 | Provide an explicit statement of the objective(s) or question(s) the review addresses. |  |
| **METHODS** | | |  |
| Eligibility criteria | 5 | Specify the inclusion and exclusion criteria for the review and how studies were grouped for the syntheses. |  |
| Information sources | 6 | Specify all databases, registers, websites, organisations, reference lists and other sources searched or consulted to identify studies. Specify the date when each source was last searched or consulted. |  |
| Search strategy | 7 | Present the full search strategies for all databases, registers and websites, including any filters and limits used. |  |
| Selection process | 8 | Specify the methods used to decide whether a study met the inclusion criteria of the review, including how many reviewers screened each record and each report retrieved, whether they worked independently, and if applicable, details of automation tools used in the process. |  |
| Data collection process | 9 | Specify the methods used to collect data from reports, including how many reviewers collected data from each report, whether they worked independently, any processes for obtaining or confirming data from study investigators, and if applicable, details of automation tools used in the process. |  |
| Data items | 10a | List and define all outcomes for which data were sought. Specify whether all results that were compatible with each outcome domain in each study were sought (e.g. for all measures, time points, analyses), and if not, the methods used to decide which results to collect. |  |
|  | 10b | List and define all other variables for which data were sought (e.g. participant and intervention characteristics, funding sources). Describe any assumptions made about any missing or unclear information. |  |
| Study risk of bias assessment | 11 | Specify the methods used to assess risk of bias in the included studies, including details of the tool(s) used, how many reviewers assessed each study and whether they worked independently, and if applicable, details of automation tools used in the process. |  |
| Effect measures | 12 | Specify for each outcome the effect measure(s) (e.g. risk ratio, mean difference) used in the synthesis or presentation of results. |  |
| Synthesis methods | 13a | Describe the processes used to decide which studies were eligible for each synthesis (e.g. tabulating the study intervention characteristics and comparing against the planned groups for each synthesis (item #5)). |  |
|  | 13b | Describe any methods required to prepare the data for presentation or synthesis, such as handling of missing summary statistics, or data conversions. |  |
|  | 13c | Describe any methods used to tabulate or visually display results of individual studies and syntheses. |  |
|  | 13d | Describe any methods used to synthesize results and provide a rationale for the choice(s). If meta-analysis was performed, describe the model(s), method(s) to identify the presence and extent of statistical heterogeneity, and software package(s) used. |  |
|  | 13e | Describe any methods used to explore possible causes of heterogeneity among study results (e.g. subgroup analysis, meta-regression). |  |
|  | 13f | Describe any sensitivity analyses conducted to assess robustness of the synthesized results. |  |
| Reporting bias assessment | 14 | Describe any methods used to assess risk of bias due to missing results in a synthesis (arising from reporting biases). |  |
| Certainty assessment | 15 | Describe any methods used to assess certainty (or confidence) in the body of evidence for an outcome. |  |
| **RESULTS** | | |  |
| Study selection | 16a | Describe the results of the search and selection process, from the number of records identified in the search to the number of studies included in the review, ideally using a flow diagram. |  |
|  | 16b | Cite studies that might appear to meet the inclusion criteria, but which were excluded, and explain why they were excluded. |  |
| Study characteristics | 17 | Cite each included study and present its characteristics. |  |
| Risk of bias in studies | 18 | Present assessments of risk of bias for each included study. |  |
| Results of individual studies | 19 | For all outcomes, present, for each study: (a) summary statistics for each group (where appropriate) and (b) an effect estimate and its precision (e.g. confidence/credible interval), ideally using structured tables or plots. |  |
| Results of syntheses | 20a | For each synthesis, briefly summarise the characteristics and risk of bias among contributing studies. |  |
|  | 20b | Present results of all statistical syntheses conducted. If meta-analysis was done, present for each the summary estimate and its precision (e.g. confidence/credible interval) and measures of statistical heterogeneity. If comparing groups, describe the direction of the effect. |  |
|  | 20c | Present results of all investigations of possible causes of heterogeneity among study results. |  |
|  | 20d | Present results of all sensitivity analyses conducted to assess the robustness of the synthesized results. |  |
| Reporting biases | 21 | Present assessments of risk of bias due to missing results (arising from reporting biases) for each synthesis assessed. |  |
| Certainty of evidence | 22 | Present assessments of certainty (or confidence) in the body of evidence for each outcome assessed. |  |
| **DISCUSSION** | | |  |
| Discussion | 23a | Provide a general interpretation of the results in the context of other evidence. |  |
|  | 23b | Discuss any limitations of the evidence included in the review. |  |
|  | 23c | Discuss any limitations of the review processes used. |  |
|  | 23d | Discuss implications of the results for practice, policy, and future research. |  |
| **OTHER INFORMATION** | | |  |
| Registration and protocol | 24a | Provide registration information for the review, including register name and registration number, or state that the review was not registered. |  |
|  | 24b | Indicate where the review protocol can be accessed, or state that a protocol was not prepared. |  |
|  | 24c | Describe and explain any amendments to information provided at registration or in the protocol. |  |
| Support | 25 | Describe sources of financial or non-financial support for the review, and the role of the funders or sponsors in the review. |  |
| Competing interests | 26 | Declare any competing interests of review authors. |  |
| Availability of data, code and other materials | 27 | Report which of the following are publicly available and where they can be found: template data collection forms; data extracted from included studies; data used for all analyses; analytic code; any other materials used in the review. |  |
